# J-shaped associations of pan-immune-inflammation value and systemic inflammation response index with stroke among American adults with hypertension: evidence from NHANES 1999–2020

**DOI:** 10.3389/fneur.2024.1417863

**Published:** 2024-07-31

**Authors:** Junchen Chen, Cheng Luo, Dianhui Tan, Yong Li

**Affiliations:** Department of Neurosurgery, the First Affiliated Hospital of Shantou University Medical College, Shantou, China

**Keywords:** systemic inflammation, stroke, NHANES, complete blood count, hypertension, smoking

## Abstract

**Introduction:**

Stroke, a leading cause of death and disability worldwide, is primarily ischemic and linked to hypertension. Hypertension, characterized by systemic chronic inflammation, significantly increases stroke risk. This study explores the association of novel systemic inflammatory markers (SII, PIV, SIRI) with stroke prevalence in hypertensive U.S. adults using NHANES data.

**Methods:**

We analyzed data from hypertensive participants in the NHANES 1999–2020 survey, excluding those under 20, pregnant, or with missing data, resulting in 18,360 subjects. Systemic inflammatory markers (SII, PIV, SIRI) were calculated from blood counts. Hypertension and stroke status were determined by self-report and clinical measurements. Covariates included sociodemographic, lifestyle, and medical history factors. Weighted statistical analyses and multivariate logistic regression models were used to explore associations, with adjustments for various covariates. Ethical approval was obtained from the NCHS Ethics Review Board.

**Results:**

In a cohort of 18,360 hypertensive individuals (mean age 56.652 years), 7.25% had a stroke. Stroke patients were older, had lower PIR, and were more likely to be female, single, less educated, smokers, non-drinkers, physically inactive, and have diabetes and CHD. Multivariate logistic regression showed that SII was not significantly associated with stroke. However, PIV and SIRI were positively associated with stroke prevalence. Each unit increase in lnPIV increased stroke odds by 14% (OR = 1.140, *p* = 0.0022), and lnSIRI by 20.6% (OR = 1.206, *p* = 0.0144). RCS analyses confirmed J-shaped associations for lnPIV and lnSIRI with stroke. Stratified analyses identified gender and smoking as significant effect modifiers. Smoking was significantly associated with elevated PIV, SIRI, and SII levels, especially in current smokers.

**Conclusion:**

Elevated PIV and SIRI levels significantly increase stroke prevalence in hypertensive individuals, notably among males and smokers. A predictive model with PIV, SIRI, and sociodemographic factors offers strong clinical utility.

## 1 Introduction

Stroke is an acute cerebrovascular disease in which abnormal blood supply to the brain results in impaired brain function or death (1). Strokes are mainly divided into ischemic and hemorrhagic strokes, with ischemic strokes accounting for most cases (2). Stroke is the leading cause of death and severe disability worldwide. The recent Global Burden of Disease Study 2019 report suggests that in 2019, there were 12.2 million stroke incident cases, 101 million stroke prevalent cases, 143 million disability-adjusted life years attributable to stroke, and 6.55 million stroke deaths (the second leading cause of death) worldwide (3). The absolute number of incident and prevalent cases of stroke has increased significantly over the last three decades, although age-standardized incidence, prevalence, and mortality rates have declined (but remain elevated among those under 70 years of age) (3). Stroke not only dramatically reduces patients’ quality of life, but also places a heavy economic burden on global public health. A survey by the American Heart Association put the annual direct and indirect economic burden of stroke at $45.5 billion in 2014–2015 (4). Stroke prevention and early intervention by identifying modifiable risk factors such as promoting healthy lifestyles and addressing comorbidities are key to reducing the burden of disease (5, 6).

Hypertension is one of the most common non-communicable diseases globally and represents the most significant modifiable component of all-cause morbidity and mortality (7). Hypertension is strongly associated with the development of several cardiovascular diseases (CVD), including stroke. Hypertension is one of the major modifiable risk factors for stroke, which is significantly more prevalent in hypertensive populations and is associated with considerable disability and mortality (8–10). An important hallmark of hypertension is a state of systemic chronic inflammation, a mechanism thought to be involved in the pathogenesis of stroke in hypertensive patients (11–13). Inflammatory cells including T cells and inflammatory cytokines have an important causal role in hypertension-mediated target organ damage (14). Therefore, as a shared pathophysiologic event of hypertension and stroke, the exploration of relevant systemic inflammatory markers may be of great significance for stroke prediction, screening, and prevention in hypertensive individuals.

Multiple immune cell and inflammatory markers including multiple interleukins, and tumor necrosis factor-alpha have been shown to be elevated in hypertensive patients (15). Recently, some novel markers of systemic inflammation derived from complete blood count (CBC) have been proposed. Systemic inflammatory markers are a group of biomarkers that reflect the body’s response to inflammation. A variety of conditions, including cardiovascular diseases, autoimmune diseases, and infections, can be assessed using these markers to assess inflammation, monitor disease progression, and predict outcomes. These novel systemic inflammatory markers, including systemic immune-inflammation index (SII), pan-immune-inflammation value (PIV), and systemic inflammation response index (SIRI), account for the role of CBC-derived inflammatory cells more comprehensively than the traditional neutrophil-to-lymphocyte ratio (NLR), monocyte-to-lymphocyte ratio (MLR), and platelet-to-lymphocyte ratio (PLR), and have been first proposed to be strongly correlated with prognosis in multiple cancers (16–18). Several clinical studies have shown that these systemic inflammatory markers are strongly associated with the development and clinical outcome of hypertension (19–22). However, there is still a lack of research on the association of these systemic inflammatory markers with stroke occurrence in hypertensive populations. Only two retrospective studies have suggested that SII and SIRI are associated with the risk of stroke in hypertensive individuals, and both were relatively small cohorts from Asia (23, 24). There is a dearth of large sample and representative studies from other countries/regions. Furthermore, whether PIV is also a predictor of stroke in hypertensive populations remains unknown.

In this study, we aimed to explore the association of SII, PIV, and SIRI with stroke prevalence in hypertensive subjects using a U.S. nationally representative population-based cross-sectional survey, the National Health and Nutrition Examination Survey (NHANES). These findings may help to reveal the predictive value of these novel systemic inflammatory markers for stroke in hypertensive adults in the U.S. and provide a rationale for stroke prevention.

## 2 Methods

### 2.1 Study design and population

NHANES is a national survey conducted by the National Center for Health Statistics (NCHS) to assess the health and nutritional status of noninstitutionalized children and adults in the U.S. NCHS is part of the Centers for Disease Control (CDC) and is responsible for providing vital health epidemiologic data for the nation. NHANES is a nationally representative cross-sectional survey with multi-stage cluster probability sampling that includes questionnaires from comprehensive household interviews and physical examination data administered by professionals (25, 26).

**Figure 1:**
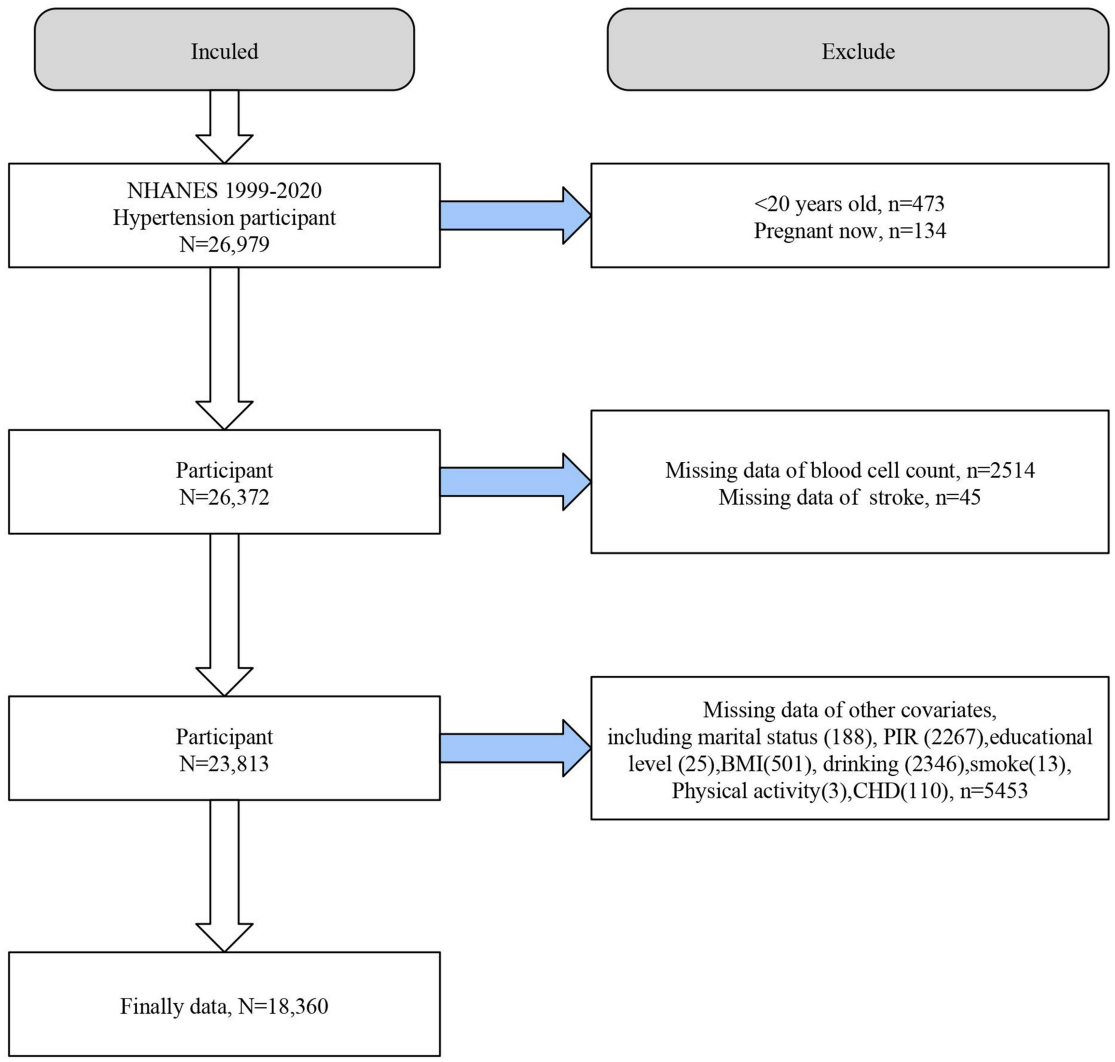
Flowchart of the study population. BMI,body mass index. CHD, coronary heart disease. NHANES, the National Health and Nutrition Examination Survey. PIR, poverty income ratio.

We first included all hypertensive participants in NHANES 1999–2020 (*n* = 26,979) and excluded individuals <20 years of age (*n* = 473), pregnant individuals (*n* = 134), those with missing blood count data (*n* = 2,514), missing stroke diagnostic information (*n* = 45), and missing covariates (*n* = 5,453). Ultimately, 18,360 individuals with hypertension were included in further analyses (Figure 1).

### 2.2 Calculation of SII, PIV, and SIRI

Beckman Coulter DxH 800 instrument at NHANES Mobile Examination Center was used to determine neutrophil, lymphocyte, monocyte, and platelet counts (expressed as × 103 cells/μl). SII = (platelet count × neutrophil count)/lymphocyte count; PIV = (platelet count × neutrophil count × monocyte count)/lymphocyte count; SIRI = (neutrophil count × monocyte count)/lymphocyte count. Since SII, PIV, and SIRI were skewed in the study population, we performed a natural logarithmic (ln) transformation of their values.

### 2.3 Hypertension diagnosis

Hypertensive status was determined by a self-reported history of hypertension, a blood pressure test that indicated hypertension (≥ 140/90 mmHg) or taking anti-hypertensive medications (20). Blood pressure testing is performed by professional technicians at the Mobile Examination Center (MEC) using mercury sphygmomanometers or electronic blood pressure measuring devices (NHANES 2017–2020) for three consecutive systolic and diastolic blood pressure measurements and in accordance with relevant international guidelines (27).

### 2.4 Stroke assessment

Participants’ stroke status was determined by self-reported medical history in NHANES. Participants who answered “yes” to the question “Has a doctor or other health professional ever told you that you had a stroke?” were considered stroke survivors.

### 2.5 Covariates

Participants’ sociodemographic characteristics, lifestyle, and medical comorbidity information were collected. Sociodemographic variables include age, gender, race/ethnicity, education level, family income-poverty ratio (PIR), and marital status, which were obtained through self-reporting in the NHANES demographic file. Lifestyle variables include body mass index (BMI), smoking, alcohol consumption, and physical activity. BMI = body weight (kg) divided by height (m) squared. Smoking status was categorized into current (active) smokers and inactive smokers (namely never smokers and former smokers) and was determined based on whether participants self-reported a lifetime number of cigarettes of at least 100 and whether they were currently smoking. Drinking history was categorized as never drinkers, former drinkers, light, moderate and heavy drinkers based on previous studies (28). Physical activity intensity was categorized as never, moderate, and vigorous based on self-reports from participants in the Global Physical Activity Questionnaire (29). The history of medical comorbidities includes diabetes and coronary heart disease (CHD). Diabetes status was determined by self-report, glucose/glucose tolerance testing, and history of relevant medication use (30). CHD history was obtained by self-report.

### 2.6 Statistical analysis

We weighted the statistical analyses according to the weighting method recommended by the NHANES Analytic Guidelines (WTMEC2YR) to ensure that the study sample was nationally representative. Data were processed and analyzed using R version 4.2.3 and EmpowerStats software. A two-sided *p* value of <0.05 was considered statistically significant. We performed baseline analyses according to stroke status in the hypertensive population (stroke and non-stroke groups), continuous variables were analyzed for between-group differences using weighted t tests and expressed as mean and standard error, and categorical variables were analyzed using weighted chi-square tests and expressed as number (percentage). Multivariate logistic regression models with varying degrees of adjustment were used to explore the association of SII, PIV, and SIRI with stroke in hypertensive individuals. Model 1 did not adjust for any covariates; Model 2 was a partial adjusted model, adjusting for age, gender, race, PIR, education level, and marital status; and Model 3 was a fully adjusted model, adjusting for all covariates. Restricted cubic spline (RCS) analysis was used to discuss potential nonlinear correlations. The curve-fitting term was defined by the RCS function in the rms package, and the degrees of freedom (or knots) were determined according to the magnitude of the nonlinearity *p* value. The correlation matrix analysis was applied in this study to explore the relationship between SII, PIV, and SIRI. Stratified analyses were performed to explore whether these associations remained consistent across subgroups and to identify potential effect modifiers. The univariate analysis was conducted to assess the relationship between potential effect modifier and elevated levels of SII, PIV, and SIRI.

### 2.7 Ethics statement

All NHANES datasets included in this study were reviewed and approved by the NCHS Ethics Review Board, and all subjects provided written informed consent. This study analyzed pre-existing public datasets and all participants were de-identified and anonymized, hence ethical review approval from local institutions was waived.

## 3 Results

### 3.1 Baseline analysis

Baseline analyses according to stroke status in the hypertensive population were presented in Table 1. A total of 18,360 hypertensive individuals were included (mean age 56.652 years), with a stroke prevalence of 7.25%. Compared with participants without stroke, the stroke population was older, had a lower PIR, and was more likely to be female, single, ≤high school educated, quitters/current smokers, never drinkers/abstainers, physically inactive, and to have diabetes and CHD. Lymphocyte counts did not differ between groups, whereas significant differences existed in monocyte, neutrophil, and platelet counts. Interestingly, we found that lnSII was not different between groups (*p* = 0.077), whereas lnPIV and lnSIRI were significantly higher in stroke patients (*p* < 0.0001 for both).

**Table 1:** Survey-weighted baseline characteristics of individuals with hypertension from NHANES 1999–2020.

	Total	No-stroke	Stroke	*p* value
*N*	**18,360**	**17,028**	**1,332**	
Representing individuals	**71,361,366**	**67,325,864**	**4,035,502**	
Age	56.652 ± 0.214	56.085 ± 0.217	66.118 ± 0.456	<0.0001
PIR	3.003 ± 0.031	3.041 ± 0.031	2.368 ± 0.066	<0.0001
BMI, kg/m^2^	30.837 ± 0.080	30.864 ± 0.082	30.396 ± 0.272	0.09
Lymphocyte, 1000 cells/μl	2.153 ± 0.017	2.157 ± 0.017	2.084 ± 0.046	0.144
Monocyte, 1000 cells/μl	0.589 ± 0.003	0.587 ± 0.003	0.618 ± 0.008	<0.001
Segmented neutrophils, 1000 cells/μl	4.418 ± 0.022	4.410 ± 0.023	4.549 ± 0.060	0.025
Platelet, 1000 cells/μL	253.957 ± 1.014	254.493 ± 0.987	245.010 ± 3.430	0.004
SII	587.718 ± 4.276	585.218 ± 4.300	629.437 ± 17.283	0.012
Ln (SII)	6.228 ± 0.007	6.226 ± 0.007	6.264 ± 0.020	0.077
PIV	353.577 ± 3.543	351.382 ± 3.580	390.204 ± 11.182	<0.001
Ln (PIVI)	5.642 ± 0.009	5.638 ± 0.010	5.717 ± 0.026	0.003
SIRI	1.379 ± 0.012	1.366 ± 0.012	1.587 ± 0.043	<0.0001
Ln (SIRI)	0.143 ± 0.009	0.136 ± 0.009	0.261 ± 0.024	<0.0001
Sex				<0.001
Male	9,193 (49.149)	8,538 (49.499)	655 (43.312)	
Female	9,167 (50.851)	8,490 (50.501)	677 (56.688)	
Race				0.135
Mexican American	2,472 (5.237)	2,341 (5.321)	131 (3.851)	
Non-Hispanic Black	4,583 (12.330)	4,212 (12.212)	371 (14.294)	
Non-Hispanic White	8,623 (72.657)	7,942 (72.648)	681 (72.797)	
Other Hispanic	1,312 (4.354)	1,235 (4.401)	77 (3.581)	
Other race	1,370 (5.422)	1,298 (5.418)	72 (5.478)	
Marital status				<0.0001
Non-single	10,880 (64.741)	10,180 (65.171)	700 (57.568)	
Single	7,480 (35.259)	6,848 (34.829)	632 (42.432)	
Education				<0.0001
<High school	2,336 (6.690)	2,132 (6.468)	204 (10.388)	
High school	7,282 (39.070)	6,674 (38.563)	608 (47.525)	
>High school	8,742 (54.240)	8,222 (54.969)	520 (42.088)	
Smoke				<0.001
Never	9,165 (49.619)	8,631 (50.070)	534 (42.100)	
Former	5,784 (31.860)	5,268 (31.598)	516 (36.233)	
Now	3,411 (18.521)	3,129 (18.332)	282 (21.666)	
Drinking				<0.0001
Never	2,760 (12.472)	2,535 (12.116)	225 (18.406)	
Former	3,827 (18.719)	3,400 (18.049)	427 (29.886)	
Mild	6,569 (37.871)	6,153 (38.195)	416 (32.466)	
Moderate	2,397 (14.818)	2,265 (15.155)	132 (9.184)	
Heavy	2,807 (16.121)	2,675 (16.484)	132 (10.057)	
Physical activity				<0.0001
No	10,290 (49.599)	9,426 (48.849)	864 (62.228)	
Moderate	4,561 (28.163)	4,267 (28.347)	294 (25.159)	
Vigorous	3,509 (22.226)	3,336 (22.804)	173 (12.613)	
Diabetes				<0.0001
No	12,933 (76.732)	12,152 (77.644)	781 (61.522)	
Yes	5,427 (23.268)	4,876 (22.356)	551 (38.478)	
Coronary heart disease				<0.0001
No	16,917 (92.774)	15,845 (93.651)	1,072 (78.149)	
Yes	1,443 (7.226)	1,183 (6.349)	260 (21.851)	

PIR, Poverty income ratio; BMI, body mass index; Ln, natural logarithmic; SII, systemic immune-inflammation index; PIV, pan-immune-inflammation value; SIRI, systemic inflammation response index. The bold values of “*N*” means Unweighted number; bold values of “Representing individuals” means Weighted number.

### 3.2 Association of SII, PIV, and SIRI with stroke prevalence in hypertensive populations

In multivariate logistic regression analyses, we found that SII was not significantly associated with the odds of stroke among hypertensive patients in all adjusted models. In fully adjusted model 3, we found that PIV was significantly and positively associated with the prevalence of stroke in the hypertensive population. Each unit increase in lnPIV was associated with a 14% increase in the odds of stroke (odds ratio [OR] = 1.140, 95% CI = 1.048–1.241, *p* = 0.0022). Higher PIV was significantly associated with an increased prevalence of stroke (p for trend = 0.0056), and hypertensive patients with PIV at Q4 had significantly increased odds of stroke compared to Q1 (OR and 95% CI = 1.235 (1.048, 1.456), *p* = 0.012). Similarly, SIRI was positively associated with stroke prevalence after adjusting for all confounders (OR = 1.206, *p* = 0.0144). As SIRI increased, the odds of stroke increased significantly (p for trend = 0.0244). Interestingly, SIRI was significantly inversely associated with stroke prevalence only at Q2 (OR = 0.767, *p* = 0.0456) (Table 2).

**Table 2:** Survey-weighted logistic regression examining the association of SII, PIV, SIRI with the prevalence of stroke among individuals with hypertension from NHANES 1999–2020.

	Model 1	Model 2	Model 3
SII	1.000 (1.000, 1.000) 0.0080	1.000 (1.000, 1.000) 0.0458	1.000 (1.000, 1.000) 0.1076
SII Log	1.136 (0.986, 1.309) 0.0785	1.108 (0.963, 1.275) 0.1533	1.078 (0.933, 1.244) 0.3102
SII quartile			
Q1	Ref.	Ref.	Ref.
Q2	1.120 (0.880, 1.425) 0.3580	1.200 (0.926, 1.557) 0.1699	1.189 (0.907, 1.559) 0.2127
Q3	0.853 (0.666, 1.092) 0.2093	0.893 (0.685, 1.164) 0.4032	0.868 (0.662, 1.140) 0.3108
Q4	1.210 (0.978, 1.497) 0.0811	1.197 (0.946, 1.516) 0.1366	1.154 (0.905, 1.473) 0.2502
P for trend	0.3011	0.4612	0.6994
PIV	1.000 (1.000, 1.001) 0.0011	1.000 (1.000, 1.000) 0.0043	1.000 (1.000, 1.000) 0.0147
PIV Log	1.196 (1.066, 1.342) 0.0027	1.181 (1.086, 1.283) 0.0001	1.140 (1.048, 1.241) 0.0022
PIV quartile			
Q1	Ref.	Ref.	Ref.
Q2	0.797 (0.622, 1.022) 0.0759	0.932 (0.787, 1.105) 0.4204	0.925 (0.779, 1.098) 0.3709
Q3	0.973 (0.758, 1.248) 0.8275	1.008 (0.851, 1.194) 0.9244	0.979 (0.825, 1.162) 0.8092
Q4	1.251 (1.018, 1.539) 0.0347	1.311 (1.115, 1.543) 0.0011	1.235 (1.048, 1.456) 0.0120
P for trend	0.0040	0.0004	0.0056
SIRI	1.183 (1.124, 1.245) <0.0001	1.111 (1.050, 1.174) 0.0003	1.096 (1.035, 1.161) 0.0021
SIRI Log	1.431 (1.255, 1.632) <0.0001	1.255 (1.087, 1.449) 0.0023	1.206 (1.040, 1.400) 0.0144
SIRI quartile			
Q1	Ref.	Ref.	Ref.
Q2	0.787 (0.622, 0.996) 0.0475	0.782 (0.604, 1.011) 0.0626	0.767 (0.593, 0.993) 0.0456
Q3	1.071 (0.847, 1.354) 0.5699	0.976 (0.751, 1.267) 0.8539	0.930 (0.716, 1.208) 0.5883
Q4	1.571 (1.271, 1.940) <0.0001	1.292 (0.997, 1.675) 0.0547	1.211 (0.928, 1.580) 0.1603
P for trend	<0.0001	0.0048	0.0244

Model 1 did not adjust for any covariates.

Model 2 adjusted age, gender, race, PIR, education level, and marital status.

Model 3 adjusted for all covariates.

### 3.3 RCS, segmented regression and correlation matrix analysis

**Figure 2:**
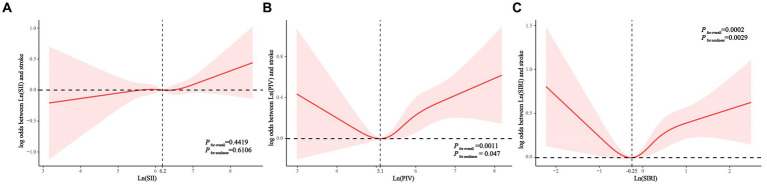
Associations of Ln(SII) **(A)**, Ln(PIV) **(B)**, and Ln(SIRI) **(C)** with the risk of stroke (presented as log odds) in hypertensive population using a restricted cubic spline regression model. PIV, pan-immune-inflammation value. SII,systemic immune-inflammation index. SIRI, systemic inflammation response index.

Consistently, RCS analyses showed that lnSII was not significantly associated with stroke prevalence in the hypertensive population (Figure 2A). Both lnPIV (p for nonlinear = 0.047) (Figure 2B) and lnSIRI (p for nonlinear = 0.0029) (Figure 2C) were nonlinearly associated with the odds of stroke and showed J-shaped associations. Segmented regression analysis indicated that lnPIV was significantly and positively associated with the prevalence of stroke in hypertensive populations at >5.1 (OR = 1.325, *p* = 0.0002) and not significantly associated before the inflection point, although p for interaction was not significant. Similarly, lnSIRI was positively associated with stroke likelihood at > − 0.25 (OR = 1.497, *p* < 0.0001) and not significantly associated before the inflection point (p for interaction = 0.0415) (Table 3).

**Table 3:** Segmented regression analysis of lnPIV and lnSIRI with the prevalence of stroke in hypertensive populations.

	Ln (SIRI) < = − 0.25	Ln (SIRI) > −0.25	P-interaction
Ln (SIRI)	0.855 (0.523, 1.399) 0.5343	1.497 (1.268, 1.768) <0.0001	0.0415
	Ln (PIV) < =5.1	Ln (PIV) > 5.1	
Ln (PIV)	1.036 (0.671, 1.599) 0.8729	1.325 (1.145, 1.533) 0.0002	0.2914

The correlation matrix among the three systemic inflammatory markers (SII, PIV, and SIRI) is as follows: There is a strong positive correlation between SII and PIV, with a correlation coefficient of 0.8634 (95% CI, 0.8596–0.8670, *p* = 0.0000), indicating a statistically significant correlation. Between SII and SIRI, there is a moderate positive correlation, with a correlation coefficient of 0.6968 (95% CI, 0.6893–0.7042, *p* = 0.0000), also statistically significant. Finally, PIV and SIRI show a strong positive correlation, with a correlation coefficient of 0.8376 (95% CI, 0.8332–0.8418, *p* = 0.0000), underscoring a statistically significant relationship (Supplementary Table S1 and Supplementary Figure S1).

### 3.4 Stratified analysis

**Figure 3:**
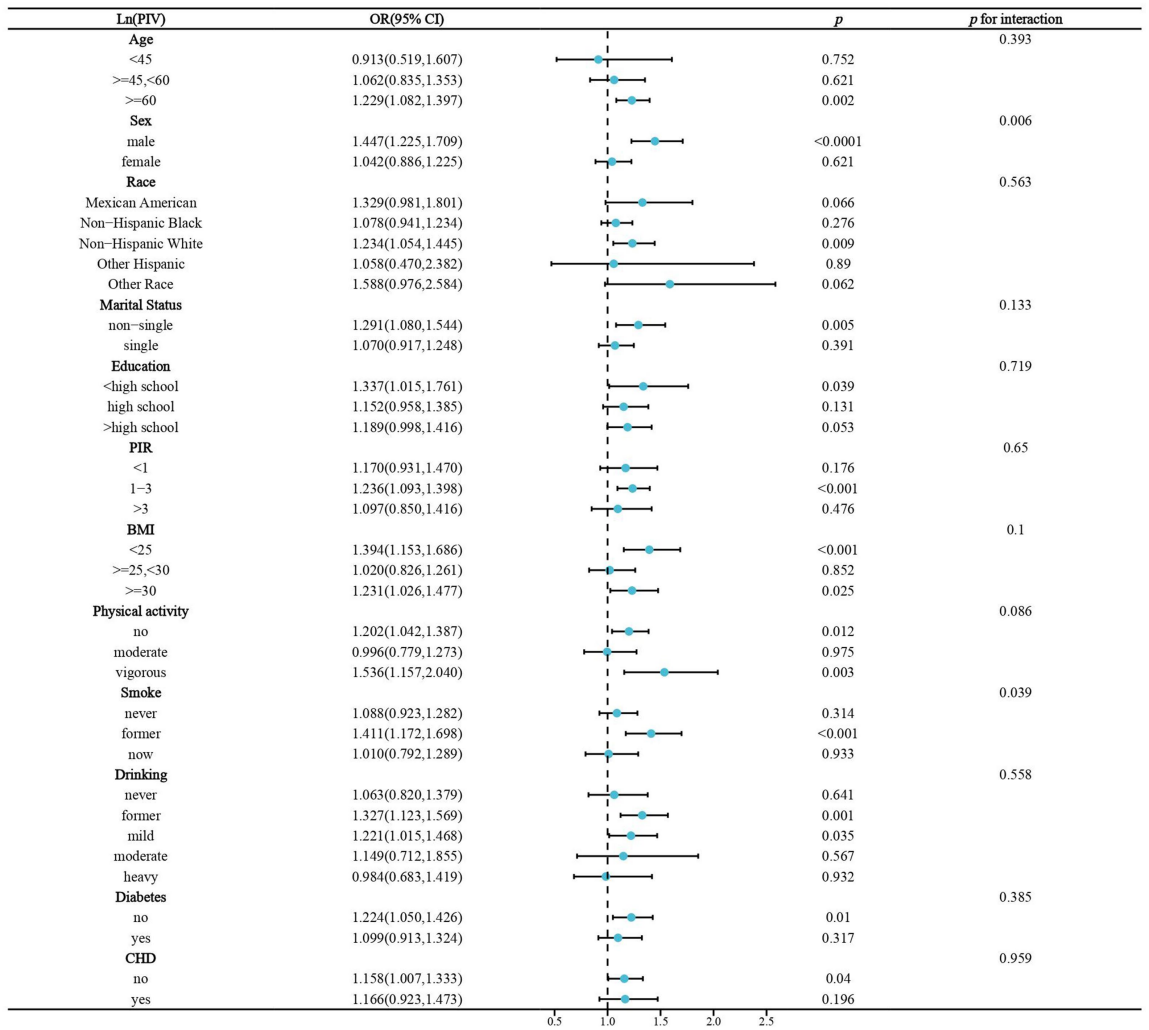
Stratified analysis of the association of Ln(PIV) and stroke prevalence in hypertensive populations. BMI,body mass index. CHD, coronary heart disease.OR, odds ratio. PIR, poverty income ratio.

**Figure 4:**
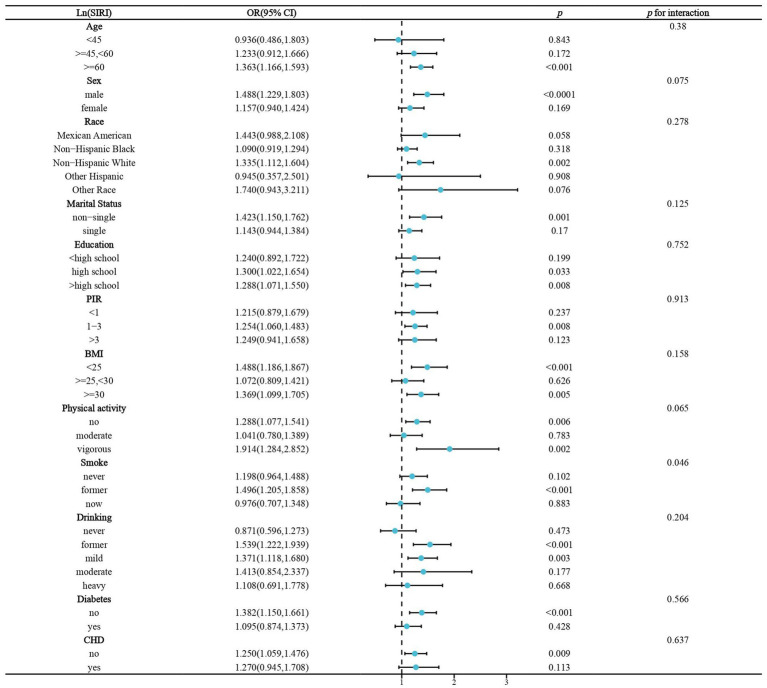
Stratified analysis of the association of Ln(SIRI) and stroke prevalence in hypertensive populations. BMI,body mass index. CHD, coronary heart disease.OR, odds ratio.PIR, poverty income ratio.

We performed stratified analyses of the association of lnPIV and lnSIRI with stroke prevalence in hypertensive populations. Significant associations between lnPIV and stroke prevalence were only present in people ≥60 years of age, men, non-Hispanic whites, non-singles, <high school diploma, PIR1-3, BMI <25 or ≥ 30 kg/m^2^, no or vigorous physical activity, quitters, abstainers/light drinkers, and those without diabetes and CHD. However, only gender and smoking were identified as significant effect modifiers (Figure 3). Subgroup analyses in the association of lnSIRI with stroke prevalence yielded broadly similar results. Smoking was the only effect modifier (Figure 4). A univariate analysis of the association between smoking and elevated levels of PIV, SIRI, and SII in individuals with hypertension was performed. For SII, former smokers have a *β* value of 16.324 (*p* = 0.0524), and current smokers have a *β* value of 26.300 (*p* = 0.0044). For PIV, former smokers have a *β* value of 31.643 (*p* < 0.0001), and current smokers have a *β* value of 57.834 (*p* < 0.0001). For SIRI, former smokers have a *β* value of 0.160 (*p* < 0.0001), and current smokers have a *β* value of 0.183 (*p* < 0.0001) (Supplementary Table S2). These results indicate a significant association between smoking and elevated levels of these indices, particularly in current smokers.

## 4 Discussion

This study represents the pioneering exploration of the relationship between SII, PIV, and SIRI levels and the incidence of stroke within a hypertensive cohort. The key findings of this investigation are outlined as follows: (1) The natural logarithm of SII (lnSII) exhibited no significant correlation with baseline clinical characteristics, suggesting its role as a relatively stable inflammation marker. Conversely, lnPIV and lnSIRI levels were notably elevated in stroke patients. (2) Within the hypertensive population, elevated levels of PIV and SIRI were strongly linked to a heightened prevalence of stroke, with the exception of SIRI, which demonstrated a significant inverse correlation with stroke prevalence solely at the second quartile (Q2). (3) Sex differences and smoking status emerged as significant effect modifiers in the relationship between lnPIV and stroke, while smoking status was the sole effect modifier in the association between lnSIRI and stroke within the hypertensive cohort.

The crucial role of chronic inflammation in stroke is well-established, and hypertension is known to related with inflammation and immunity, implying that inflammation is a common background for both diseases (31–33). In the hypertensive population, there are distinct characteristics related to inflammation and the risk of stroke. Hypertension induces various alterations in the body’s blood vessels. These changes include adaptive remodeling, hypertrophy, and stiffness of vascular walls, as well as a decrease in vessel diameter, leading to increased vascular resistance and circulatory disruptions (34, 35). The brain is particularly vulnerable to circulatory changes associated with hypertension, impacting key mechanisms that regulate cerebral blood flow and disrupt brain energy balance, ultimately affecting the function of neurovascular units (35, 36). Systemic and neuroinflammation contribute to hypertension pathophysiology by inducing reactive oxygen species (ROS) production and cell damage, triggering the release of damage-associated molecular patterns (DAMPs) that activate Toll-like receptor 4 (TLR4) (35, 37). Activation of endothelial TLR4, via MyD88 protein, stimulates transcription factors AP-1 and NF-kB, exacerbating inflammation in vascular tissues (35, 38, 39). Patients with hypertension often exhibit elevated levels of inflammatory markers, including C-reactive protein and IL-6, which have been linked to a higher risk of stroke (35, 40, 41). Immune cells in the brain, including microglia/macrophages, are impacted by hypertension, leading to their activation and expression of pro-inflammatory molecules like IL-1b, IL-6, and TNF-a. Perivascular macrophages (PVMs) near arterioles and venules play a crucial role in neurovascular unit dysfunction, with studies showing their involvement in blood–brain barrier (BBB) disruption (42, 43). CD36 receptor expression in microglia is elevated in hypertensive conditions, contributing to BBB lesions and astrocyte activation post-stroke, exacerbating inflammatory processes (44, 45). Although no studies have directly compared systemic inflammatory markers (PIV, SIRI, SII) between hypertensive and non-hypertensive patients, a study of non-dipper hypertensive patients found their SII levels to be higher than those of dipper hypertensive patients (46). Similarly, another study found a U-shaped relationship between the SII levels and the risk of hypertension in US adults, suggesting a complex role for SII in hypertension (47).

A newly proposed inflammatory biomarker, known as the Pan-Immune-Inflammation Value (PIV), incorporates neutrophil, monocyte, platelet, and lymphocyte counts to provide a more comprehensive assessment of the systemic immune inflammatory response (17, 48). The Systemic Inflammation Response Index (SIRI), comprising platelets and three subtypes of white blood cells, has been identified as a significant correlate of cardiovascular disease (CVD). Specifically, SIRI is recognized as a superior marker of chronic inflammation and demonstrates strong prognostic predictive value for patients with acute strokes and tumors (24, 49, 50). In our study, there is a strong correlation among PIV, SIRI, and SII (Supplementary Table S1 and Supplementary Figure S1). They are all similar systemic inflammation indicators derived from whole blood cells, but the composition of inflammatory cells in their respective calculation formulas differs, which may lead to some being linear and others nonlinear association between stroke prevalence. The immune and inflammatory response is a common process in the clinical manifestations of cardiac and cerebral acute ischemia following atherothrombosis (48, 51, 52). Following cerebral ischemic injury, damage-associated molecular patterns (DAMPs) released by necrotic cells trigger the activation of resident immune cells within the central nervous system, including microglia and astrocytes. This activation then leads to the recruitment of peripheral immune cells to initiate adaptive immune responses (48, 53). In our study, a robust correlation was observed between PIV, SIRI, and the prevalence of stroke among former smokers when compared to non-smokers and current smokers. Cigarette smoking elevates the risk of cardiovascular events through two primary mechanisms: an atherosclerotic effect and a prothrombotic effect. Smoking compromises vasodilation and triggers inflammation, ultimately culminating in the development of atherosclerosis (54, 55). In the recent research, Darragh Duffy from the Pasteur Institute in France, specifically examines the impact of smoking on innate and adaptive immune responses, elucidating both the short-term and long-term regulatory effects of smoking on immune reactions based on the analysis of the Milieu Intérieur cohort (56). To further assess the impact of smoking on the immune system, the authors constructed a model to evaluate the level of smoking effects. The results revealed that current smoking influences both innate and adaptive immune responses, leading to heightened inductions of CXCL5, IL-2, and IL-13. Compared to non-smokers, former smokers did not exhibit a significant increase in CXCL5 secretion following innate immune stimulation; however, the secretion of IL-2 and IL-13 increased after adaptive immune stimulation. These findings suggest that smoking exerts a short-term impact on innate immunity but has long-lasting effects on adaptive immune responses (56). The result of our study might be explained by the fact that former smoker status changed the adaptive immune responses and led to the higher level of inflammation. This suggests that the avoidance of initial exposure to tobacco is crucial for enhancing long-term immunity, thereby aiding in the reduction of stroke risk within the hypertensive population.

In the present study, a robust correlation between PIV and the prevalence of stroke in hypertension population was observed in males compared to females. The overall incidence of stroke in men is estimated to be 33% higher than in women (57). This male-predominant trend is also evident in the perinatal, neonatal, and pediatric populations, with males exhibiting an elevated risk for both hemorrhagic and ischemic strokes (58–60). Innate factors such as host genetics and chromosomal sex are pivotal in influencing both the host immune system and the neuroimmune response to brain injury. Ischemic stroke disrupts intracellular communication among astrocytes, neurons, and resident immune cells within the central nervous system (CNS). Elevated cytokine and chemokine production coordinates the recruitment of peripheral immune cells and facilitates neuroinflammation (61). Female hormones, particularly estrogen, may exert a significant role in neuroprotection following ischemic events. Estrogen, produced within the brain, functions as a neuroprotective and anti-inflammatory agent post-stroke. As a sex steroid and neurosteroid hormone, estrogen’s neuroprotective properties involve the modulation of the immunological response that arises following ischemic brain injury (62–64). Sex differences play a significant role in the occurrence and outcomes of ischemic stroke, with immune regulation based on sex being a key factor. Discrepancies in stroke incidence and prognosis may also be impacted by sex-specific risk factors, including the use of oral contraceptives and menopausal status (60, 65). Unfortunately, despite the recognition of gender disparities as a crucial risk factor in numerous studies, gender-specific stroke treatments have not yet been developed.

Our research investigated the occurrence of stroke in the hypertensive population of the United States, a demographic that has received limited attention in prior studies. This study offers important insights into the interplay between immune response, inflammation and stroke among individuals with hypertension. In our analysis, we found that the relationship between PIV and SIRI and stroke risk was non-linear, with former smokers and males experiencing an increased risk. These findings have significant implications for early identification of high-risk individuals and guiding recommendations for stroke prevention in hypertensive patients. By using these results, we can develop personalized prevention strategies, lifestyle interventions, and potential therapeutic targets to reduce inflammation in the body. Among the findings is an emphasis on the risk of stroke associated with smoking, reinforcing the importance of smoking cessation interventions and advocating for not initiating tobacco use. Moreover, these insights can assist public health policymakers in designing more effective stroke prevention programs for hypertensives, including specific interventions for high-risk groups such as men and former smokers. Considering estrogen supplementation for postmenopausal women is also suggested as a potential preventive measure. Nevertheless, our study has several limitations that warrant consideration. Firstly, a few limitations of the NHANES self-reported diagnosis include nonresponse bias, measurement errors, subjectivity, a complex survey design, and diagnostic tests that are prone to errors. A thorough consideration is needed when interpreting results from NHANES-based studies due to the possibility of biases and inaccuracies being introduced (66, 67). Secondly, it is crucial to acknowledge that our study design was cross-sectional, precluding the establishment of causal relationships between PIV, SIRI levels, and the prevalence of stroke. In our study, it can only be said that the inflammation levels in individuals with hypertension combined with stroke are higher than those without stroke in the hypertensive population, but it cannot be concluded whether inflammation is caused by hypertension or stroke. Future prospective studies are essential to validate these associations. Thirdly, despite our meticulous adjustment for potential confounding variables in the regression analysis, the influence of residual factors, such as autoimmune diseases, on the outcomes cannot be entirely eliminated. Fourthly, the NHANES database relies on standardized questionnaires during home visits to collect participants’ medical histories, limiting our ability to delve into the specific subtypes of hypertension and stroke. Consequently, the potential impact of hypertension phenotypes and stroke severity on the observed outcomes remains uncertain. Fifthly, the systemic inflammatory markers (SII, PIV, and SIRI) can potentially vary depending on the timing of blood collection. Our research has limitations regarding the specific details of blood collection timing in NHANES, but it generally involves collecting biospecimens as part of its comprehensive health assessment surveys. The specific timing of these collections could vary depending on the survey cycle and the particular health or nutritional focus of the study at any given time (68). Notably, all participants’ blood samples were collected under standardized conditions at the NHANES Mobile Examination Center (MEC). All blood samples were analyzed by professional technicians to ensure the consistency and reliability of the data (69). Furthermore, our study was confined to participants from the United States, potentially limiting the generalizability of our findings to populations with distinct risk profiles and health behaviors. Additionally, variations in treatment approaches across different generations could have influenced our results. Despite these constraints, it is imperative to recognize the need for further investigations to elucidate the roles of PIV and SIRI in the context of hypertension and stroke prevalence. Such endeavors will enhance our comprehension of the shared pathophysiological mechanisms underlying hypertension and stroke, paving the way for the development of more efficacious treatment modalities.

## 5 Conclusion

Elevated levels of PIV and SIRI in individuals with hypertension are associated with a notable rise in stroke prevalence, especially among males and former smokers. Furthermore, a predictive clinical model encompassing PIV, SIRI, age, gender, race, education, marital status, and poverty income ratio demonstrates strong prognostic utility for estimating stroke prevalence within the hypertensive population, offering valuable clinical insights.

## Data availability statement

The original contributions presented in the study are included in the article/Supplementary material, further inquiries can be directed to the corresponding author.

## Ethics statement

The studies involving humans were approved by the National Center for Health Statistics Institutional Review Board. The studies were conducted in accordance with the local legislation and institutional requirements. The participants provided their written informed consent to participate in this study. Written informed consent was obtained from the individual(s) for the publication of any potentially identifiable images or data included in this article.

## Author contributions

JC: Formal analysis, Investigation, Writing – original draft. CL: Formal analysis, Investigation, Writing – original draft. DT: Methodology, Writing – review & editing. YL: Formal analysis, Methodology, Writing – review & editing.

## Supplementary material

Supplementary Figure S1 The correlation matrix analysis among the three systemic inflammatory markers (SII, PIV, and SIRI).
