# Supplementary material for: J-shaped associations of pan-immune-inflammation value and systemic inflammation response index with stroke among American adults with hypertension: evidence from NHANES 1999–2020
Source: Front Neurol. 2024 Jul 31;15:1417863. doi: 10.3389/fneur.2024.1417863 (PMC11322096; doi:10.3389/fneur.2024.1417863)
Supplement: Supplementary file 1 [file Table_1.DOCX]

Supplementary Table S1 The correlation matrix analysis among the three systemic inflammatory markers (SII, PIV, and SIRI)

| Var 1 | Var 2 | Correlation | 95%CI low | 95%CI upp | P.value | t | df | Method |
| --- | --- | --- | --- | --- | --- | --- | --- | --- |
| SII | SII | 1.0000 | 1.0000 | 1.0000 | 0.0000 | Inf | 18358.0000 | pearson |
| SII | PIV | 0.8634 | 0.8596 | 0.8670 | 0.0000 | 231.8354 | 18358.0000 | pearson |
| SII | SIRI | 0.6968 | 0.6893 | 0.7042 | 0.0000 | 131.6262 | 18358.0000 | pearson |
| PIV | SII | 0.8634 | 0.8596 | 0.8670 | 0.0000 | 231.8354 | 18358.0000 | pearson |
| PIV | PIV | 1.0000 | 1.0000 | 1.0000 | 0.0000 | Inf | 18358.0000 | pearson |
| PIV | SIRI | 0.8376 | 0.8332 | 0.8418 | 0.0000 | 207.7151 | 18358.0000 | pearson |
| SIRI | SII | 0.6968 | 0.6893 | 0.7042 | 0.0000 | 131.6262 | 18358.0000 | pearson |
| SIRI | PIV | 0.8376 | 0.8332 | 0.8418 | 0.0000 | 207.7151 | 18358.0000 | pearson |
| SIRI | SIRI | 1.0000 | 1.0000 | 1.0000 | 0.0000 | Inf | 18358.0000 | pearson |
