# Supplementary material for: J-shaped associations of pan-immune-inflammation value and systemic inflammation response index with stroke among American adults with hypertension: evidence from NHANES 1999–2020
Source: Front Neurol. 2024 Jul 31;15:1417863. doi: 10.3389/fneur.2024.1417863 (PMC11322096; doi:10.3389/fneur.2024.1417863)
Supplement: Supplementary file 2 [file Table_2.DOCX]

Supplementary Table S2 The univariate association between smoking and each elevated levels of SII, PIV and SIRI in individuals with hypertension

| **SII** | **β (95%CI) P-value** |
| --- | --- |
| **SMOKE** |  |
| Never | Ref. |
| Former | 16.324 (-0.057, 32.706) 0.0524 |
| Now | 26.300 (8.443, 44.158) 0.0044 |
| **PIV** | **β (95%CI) P-value** |
| **SMOKE** |  |
| Never | Ref. |
| Former | 31.643 (18.893, 44.394) <0.0001 |
| Now | 57.834 (43.653, 72.014) <0.0001 |
| **SIRI** | **β (95%CI) P-value** |
| **SMOKE** |  |
| Never | Ref. |
| Former | 0.160 (0.119, 0.201) <0.0001 |
| Now | 0.183 (0.134, 0.231) <0.0001 |
