# Supplementary figures and images for: J-shaped associations of pan-immune-inflammation value and systemic inflammation response index with stroke among American adults with hypertension: evidence from NHANES 1999–2020
Source: Front Neurol. 2024 Jul 31;15:1417863. doi: 10.3389/fneur.2024.1417863 (PMC11322096; doi:10.3389/fneur.2024.1417863)

SIRI

SII

PIV

\*\*\*

\*\*\*

\*\*\*

SIRI

\*\*\*

\*\*\*

SII

\*\*\*

PIV

\*  $p < 0.05$ \*\*  $p < 0.01$ \*\*\*  $p < 0.001$ 

Cor

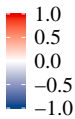

Supplement: Supplementary file 3 [file Data_Sheet_1.PDF]
